# Body composition as an indicator of metabolic changes in mice obtained by in vitro fertilization

**DOI:** 10.18699/VJGB-23-43

**Published:** 2023-07

**Authors:** M.V. Anisimova, Yanli Gon, G.V. Kontsevaya, A.V. Romashchenko, N.V. Khotskin, A.K. Stanova, L.A. Gerlinskaya, M.P. Moshkin1

**Affiliations:** Institute of Cytology and Genetics of the Siberian Branch of the Russian Academy of Sciences, Novosibirsk, Russia; Institute of Cytology and Genetics of the Siberian Branch of the Russian Academy of Sciences, Novosibirsk, Russia; Institute of Cytology and Genetics of the Siberian Branch of the Russian Academy of Sciences, Novosibirsk, Russia; Institute of Cytology and Genetics of the Siberian Branch of the Russian Academy of Sciences, Novosibirsk, Russia; Institute of Cytology and Genetics of the Siberian Branch of the Russian Academy of Sciences, Novosibirsk, Russia; Institute of Cytology and Genetics of the Siberian Branch of the Russian Academy of Sciences, Novosibirsk, Russia; Institute of Cytology and Genetics of the Siberian Branch of the Russian Academy of Sciences, Novosibirsk, Russia; Institute of Cytology and Genetics of the Siberian Branch of the Russian Academy of Sciences, Novosibirsk, Russia Tomsk State University, Department of Vertebrate Zoology and Ecology, Tomsk, Russia

**Keywords:** in vitro fertilization, mature offspring, epigenetic transformation, body composition, feed consumption, glucose tolerance, фертилизация in vitro, половозрелые потомки, эпигенетическая трансформация, композиция тела, потребление корма, глюкозотолерантность

## Abstract

To identify body systems subject to epigenetic transformation during in vitro fertilization (IVF), comparative morphological and functional studies were performed on sexually mature offspring of outbred CD1 mice, specific-pathogen-free (SPF), obtained by IVF (experiment) and natural conception (control). The studies included assessment of age-related changes in body weight and composition, energy intake and expenditure, and glucose homeostasis. To level the effects caused by the different number of newborns in the control and in the experiment, the size of the fed litters was halved in the control females. Males obtained using the IVF procedure were superior in body weight compared to control males in all age groups. As was shown by analysis of variance with experiment/control factors, gender, age (7, 10 and 20 weeks), the IVF procedure had a statistically significant and unidirectional effect on body composition. At the same time, IVF offspring outperformed control individuals in relative fat content, but were behind in terms of lean mass. The effect of the interaction of factors was not statistically significant. IVF offspring of both sexes had higher fat to lean mass ratios (FLR). Since adipose tissue contributes significantly less to total energy intake compared to muscle, the main component of lean mass, it is not surprising that at the same level of IVF locomotor activity offspring consumed less food than controls. When converted to one gram of body weight, this difference reached 19 %. One of the consequences of reduced utilization of IVF energy substrates by offspring is a decrease in their tolerance to glucose loading. The integral criterion for the effectiveness of restoring the initial glucose level is the area under the curve (AUC), the value of which was 2.5 (males) and 3.2 (females) times higher in IVF offspring compared to the corresponding control. Thus, the totality of our original and literature data shows an increase in the risk of metabolic disorders in IVF offspring, which is confirmed by epidemiological studies of a relatively young cohort of people born using assisted reproductive technologies.

## Abstract (ru)

Для выявления систем организма, подверженных эпигенетической трансформации при оплодотворении in vitro (IVF), были выполнены сравнительные морфофункциональные исследования половозрелых потомков мышей аутбредной линии CD1, свободных от патогенов (SPF-статус), полученных путем IVF (опыт) и при естественном зачатии (контроль). Исследования включали в себя оценку возрастной динамики массы и композиций тела, потребления и расходования энергии, а также глюкозный гомеостаз. Для нивелирования эффектов, обусловленных разным числом новорожденных в контроле и в опыте, у контрольных самок сокращали вдвое размеры выкармливаемых пометов. Самцы, полученные с использованием процедуры IVF, превосходили по массе тела контрольных самцов во всех возрастных группах. Как показал дисперсионный анализ с факторами «опыт/контроль», «пол», «возраст» (7, 10 и 20 недель), процедура IVF статистически значимо и однонаправленно влияла на композиционный состав тела. При этом IVF потомки превосходили контрольных особей по относительному содержанию жира, но проигрывали им по значениям тощей массы. Эффект взаимодействия факторов был статистически не значимым. У IVF потомков обоего пола были отмечены большие значения отношений жира к тощей массе (FLR). Поскольку жировая ткань вносит значительно меньший вклад в общее потребление энергии по сравнению с мышцами – основным компонентом тощей массы, то неудивительно, что при одинаковом уровне двигательной активности IVF потомки потребляли меньше корма, чем контрольные животные. При пересчете на один грамм массы тела эта разница достигала 19 %. Одним из следствий пониженной утилизации энергетических субстратов IVF потомками является снижение их толерантности к нагрузке глюкозой. Интегральным критерием эффективности восстановления исходного уровня глюкозы служит площадь под кривой (AUC), величина которой была в 2.5 раза (самцы) и 3.2 раза (самки) выше у IVF потомков по сравнению с соответствующим контролем. Таким образом, совокупность собственных и литературных данных показывает увеличение риска метаболических нарушений у IVF потомков, что подтверждают эпидемиологические исследования сравнительно молодой когорты людей, рожденных с применением вспомогательных репродуктивных технологий

## Introduction

One of the civilizational problems significantly affecting the health of new generations is the increasing spread of assisted reproductive technologies (ART), including in vitro fertilization (IVF), as well as intracytoplasmic sperm injection (ICSI), in vitro cultivation of preimplantation embryos and embryo transfer to surrogate mothers. In the 44 years since the first successful IVF pregnancy, the number of people conceived in vitro has exceeded 10 million and accounts for about 2 % of all newborns in developed countries (Wyns et al., 2018). In Russia, their number exceeds 130,000, of whom 90,000 have been born in the last five years, and the percentage of successful pregnancies with the use of ART has now increased approximately threefold (Russian Association…, 2019).

Despite the youthfulness of the generation of people born with ART, this group of offspring has a higher risk of diabetes, metabolic disorders, arterial hypertension, and neuropsy-chiatric disorders, compared to those observed in the sameage groups of offspring of natural conception (Hart, Norman, 2013; Hyrapetian et al., 2014; Duranthon, Chavatte-Palmer, 2018; Halliday et al., 2019). These results allow us to predict a more rapid development of age-related pathologies, which may become a real public health problem. According to data from specialized clinics, IVF is used not only by couples where the inability to conceive is due to the age of one or both partners, but also by patients with health disorders, in particular overweight, disorders of psycho-emotional status and other diseases (Cauldwell et al., 2017; Farquhar et al., 2019). Therefore, based on clinical observations alone, it is difficult to differentiate between the contribution to potential health impairments of the IVF procedure itself and the genetic and physiological characteristics of the parents.

The most adequate approach to assess the phenotypic effects of IVF and ART is experimental studies performed under controlled conditions on standardized laboratory animals. It is the experiment that can reveal the pros and cons of in vitro fertilization in solving the demographic problems of modern society. It should be noted that the experimental data available in the literature support the phenotypic significance of IVF (Roy et al., 2017; La Rovere et al., 2019). One of the actively developed aspects of phenotypic modulation of offspring born with ART refers to the increased risk of metabolic abnormalities (Heber, Ptak, 2020). Experiments on laboratory mice have provided evidence of an independent role of IVF in the formation of metabolic syndrome and obesity (Feuer et al., 2014), body composition (Sjöblom et al., 2005), and changes in carbohydrate homeostasis and predisposition to diabetes (Scott et al., 2010). However, these effects of IVF have not been confirmed in all studies and vary depending on the sex of the animals and conditions of embryo development outside the maternal body (Donjacour et al., 2014). At the same time, the question of the key factors that determine the manifestation of the metabolic syndrome in adult offspring obtained by in vitro fertilization remains out of sight.

Metabolic syndrome is a combination of hyperglycemia, abdominal obesity, dyslipidemia, and hypertension, and their manifestation is determined by eating behavior, physical activity, and food intake (Sousa, Norman, 2016). The most important factor leading to the development of metabolic syndrome is a change in the balance between energy expenditure, and its compensation by food calories. Moreover, fat accumulation is determined not only by the amount of food consumed, but also by its distribution in the daily cycle (Gill, Panda, 2015). At the same time, the analysis of the eating behavior of IVF mice is found in sporadic studies and is limited to the estimation of daily feed intake without analyzing circadian dynamics and without comparing it with the level of locomotor activity (Feuer et al., 2014).

Since the above-mentioned deviations of individual development are interrelated, it is of fundamental importance to investigate them comprehensively within a single experiment. But, as a rule, these works are limited to the study of individual phenotypic characteristics at different stages of individual development, which makes it difficult to analyze the causeeffect relationships between successive ontogenetic events.

In our work, we investigated the effect of ART on the formation of interdependencies of indicators of daily activity dynamics, feed intake, glucose homeostasis, and body composition associated with the risk of metabolic syndrome in naturally conceived and IVF-obtained sexually mature CD1 line mice progeny. The CD1 line mice do not have their own unique MHC haplotype (Marín et al., 2014) and this circumstance allows us to exclude the influence of the MHC haplotype differences between the embryos and the gestating mother on embryo development during pregnancy and, consequently, on the phenotype of adult progeny (Gerlinskaya, Evsikov, 2001; Rapacz-Leonard et al., 2014). We showed that IVF-obtained progeny of CD1 line are characterized by excess body weight, which is combined with an increase in the relative proportion of fat and with reduced tolerance to glucose load.

## Materials and methods

The study was performed in the Center for Genetic Resources of Laboratory Animals of the Federal Research Center Institute of Cytology and Genetics, Siberian Branch of the Russian Academy of Sciences, on outbred CD1 mice free from species- specific pathogens (SPF status). SPF status compliance was confirmed by pathogen analysis according to the European Laboratory Animal Health Association (FELASA) list.

The animals were kept in individually ventilated OptiMice cages (USA). The controlled environmental conditions had the following parameters: photoperiod 14C:10T, temperature 22–24 °C and humidity 40–50 %. Gradual switching off of the light began at 16:00 local time. Dusted birch pellets (Albion LLC, Novosibirsk) were used as bedding material. Food (SNIFF, Germany) and water were given without restrictions. Feed and bedding were given to animals after autoclaving (121 °C).

Experimental groups. IVF group – males and females obtained using in vitro fertilization (IVF), cultivation and embryo transfer; Control group – males and females obtained as a result of natural mating.

Offspring: in vitro fertilization, culture conditions and embryo transfers. IVF procedures, embryo culture conditions and embryo transfers were performed according to the technique described previously (Kontsevaya et al., 2021). IVF oocytes were obtained from female CD1 mice after superovulation by intraperitoneal injection of 5 IU of pregnant mare serum gonadotropin (PMSG) (Intervet International, Netherlands) followed by 5 IU of human chorionic gonadotropin (chorulon) (Intervet International, Netherlands) at 48 h intervals. Cumulus-oocyte complexes collected from the oocyte ampoule 17–18 h after hCG injection were placed in a drop (200 μl) of HTF medium (Human Tubal Fluid, Irvine Scientific, USA) for fertilization. To obtain spermatozoa, the caudal part of the epididymis was placed in HTF medium, and after incubation (1 h), a 3–5 μl drop containing spermatozoa was added to the oocytes and incubated for 4–5 h. Fertilized oocytes were washed in four drops of HTF medium and cultured for 72 h in 60 μl KSOM medium 8–12 embryos per drop under mineral oil (Sigma), at 37 °C and 5 % CO2 until the blastocyst stage. The developmental efficiency of the embryos after IVF was 75.5 ± 2.86 % at 2 cells stage and 70.6 ± 4.64 % from 2 cells stage to blastocysts.

Blastocysts were transferred to CD1 pseudopregnant females on day 2.5 of pseudopregnancy (Kontsevaya et al., 2021). Pseudopregnancy was induced by mating females with vasectomized males of the same line. Male vasectomies were performed by thermal cauterization of the vas deferens at least 2 weeks before mating. Surgical procedures were performed under general anesthesia (Domitor, Orion Pharma, Finland – 15 μg/100 g body weight and Zoletil, Virbac, France – 3 mg/100 g body weight, intraperitoneally). The morning after mating, the females were examined and, in the presence of vaginal plugs, transferred to separate cages. On day 2.5 of pseudopregnancy, 17 females were surgically transferred 12 blastocysts each into the left oviduct under gas anesthesia (Aerrane, Baxter Healthcare Corp., USA). After embryonic transfers, the females were placed in separate cages until delivery. Since, as previously shown, the surgical procedures performed in embryonic transfers (narcotization and introduction of culture medium into the uterus) did not affect the course of pregnancy and hormonal background (Gerlinskaya, Evsikov, 2001), therefore, control males were obtained by natural mating.

After IVF procedures and embryo transfers, 13 females (76.5 %) gave birth and all newborn offspring were fed without losses. The offspring after maternal feeding (3 weeks) were weaned from the mothers and further kept in single-sex groups of 5 individuals in each cage. The average number of nursed offspring produced by IVF was 3.6 ± 0.24 and was significantly lower compared to 12.5 ± 0.58 in natural pregnancy. The decrease in litter size is caused by transplanting blastocysts into only one uterine horn, whereas in a natural pregnancy, fetuses develop in two horns. In turn, litter size affects maternal behavior and offspring development (Enes- Marques, Giusti-Paiva, 2018). To compensate for the effects of the number of nursed offspring, females in the control group had some of their newborns removed and the number of nursed offspring was reduced by 4–5 individuals per litter. Offspring from 13 IVF litters and 8 reduced control litters were examined (Table 1).

**Table 1.**
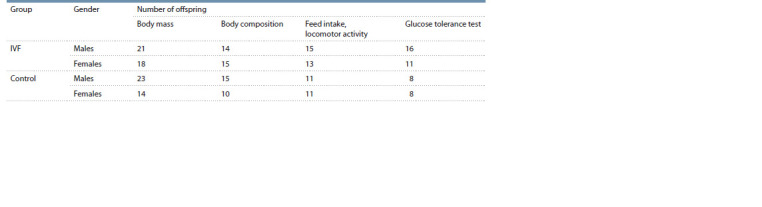
Progeny studied

Body mass and body composition of the offspring were determined at 7–8, 10–11, and 19–20 weeks of age. Fat and lean mass were quantified using a low-field NMR (nuclear magnetic resonance) spectrometer EchoMRI (USA).

Locomotor activity and feed consumption were measured in males and females of the control and experimental groups at 11–12 weeks of age. Animals were housed one at a time in Phenomaster cages (TSE, Germany). After a 2-day period of habituation, we measured the distance traveled (spontaneous locomotor activity), water and feed consumption for 3 days. When analyzing circadian rhythms of locomotor and food activity, the values of the analyzed parameters were summed at 1-hour intervals. For intergroup comparisons of locomotor activity, feed and water consumption, we used the total values for the 3-day observation period.

Glucose tolerance test (GTT) was performed according to the standard technique on males and females of control and experimental groups at the age of 10–11 weeks. 16 hours before glucose injection, the feeder was removed from the mice maintenance cages. Glucose (PanEco, Russia) was injected intraperitoneally at the rate of 10 μl 20 % glucose per 1 g mouse weight. Blood was taken from the tip of the tail at five time points: 0 – baseline level before intraperitoneal injection of glucose solution, 1 – after 15 min, 2 – after 30 min, 3 – after 60 min, and 4 – after 120 min after intraperitoneal injection of glucose solution. Blood glucose levels were measured using a Contour TS glucose meter (Bayer, Switzerland). The area under the curve of increase from the baseline glucose concentration level (average under curve – AUC) was calculated by numerical integration as an integral index of GGT.

Statistical analysis was started by assessing the nature of the distribution of empirical data. According to the Kolmogorov– Smirnov criterion, all variation series of the analyzed traits corresponded to normal distribution. Therefore, two- or three-factor analysis of variance and analysis of variance with repeated measures were used to determine the effects of experimental group, age, and sex. Comparisons of the 2 mean values were performed using Student’s test (t-test). Data are presented as means and errors (mean ± SE).

## Results

Mass and body composition

**Fig. 1.**
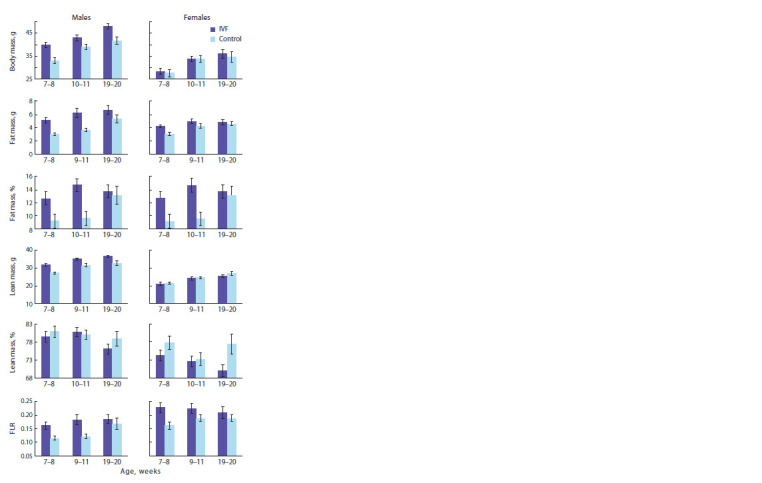
Mass and body composition in IVF and control groups at different ages. FLR is the ratio of fat to lean.

Analysis of variance with the factors of experiment/control, sex, and age (7–8, 10–11, and 19–20 weeks) showed that the IVF procedure had a statistically significant effect on body weight and composition (Table 2, Fig. 1).

**Table 2.**
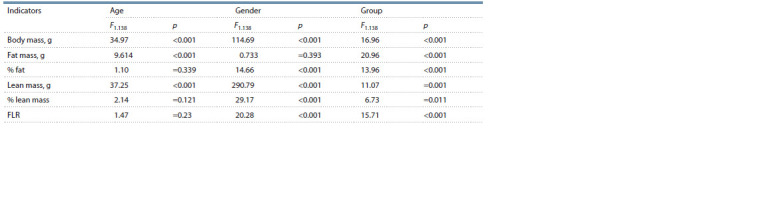
Age, gender and experimental group effects on body mass and body composition (two-factor analysis of variance) Notе. The effects of factor interactions were statistically unreliable and therefore are not included in the table

There was no interaction of factors for any of the analyzed traits, indicating a unidirectional effect of in vitro fertilization in different sex and age groups. Analysis of the effects of IVF performed separately for males and females revealed statistically significant differences in body weight between control and experiment only in males whose IVF progeny outweighed (43.7 ± 0.7 g) the control (38.0 ± 0.8 g) in all age groups (from 3 to 20 weeks). The statistical significance of IVF was confirmed by a two-factor analysis of variance with control/experiment and age factors (F1,77 = 28.6, p <0.001). The same technique was applied to analyze the effects of IVF on body composition in males and females. Total fat and lean mass were higher in IVF males (6.0 ± 0.3 and 34.2 ± 0.4 g, respectively) than in controls (4.0 ± 0.3 and 30.3 ± 0.4 g; F1,77 = 20.5, p < 0.001 and F1,77 = 40.0, p < 0.001). In females, IVF had a statistically significant effect only on fat content: IVF, 5.1 ± 0.2 g, control, 4.4 ± 0.3 g; F1,61 = 4.4, p = 0.04.

The effects listed above are due in part to the effect of IVF on animal body weight. But intergroup differences (IVF vs control) persisted when analyzing relative body composition indices. Thus, the percentage of fat in IVF males (13.4 ± 0.6 %) and females (15.7 ± 0.5 %) exceeded that of controls (males, 10.6 ± 0.7 %; F1,77 = 10.5, p = 0.002; females, 13.6 ± 0.8 %; F1,61 = 4.7, p = 0.03). In contrast to fat, the proportion of lean mass was higher in controls, but the effect of IVF was statistically significant only in females: control, 76.2 ± 1.3 %, IVF, 72.4 ± 0.9 %; F1,61 = 5.8, p = 0.02.

FLR also depended on sex and experimental group. In this case, males and females obtained by in vitro fertilization surpassed control individuals in FLR: IVF males, 0.175 ± 0.008 and control, 0.133 ± 0.009; F1,77 = 10.2, p = 0.002; IVF females, 0.221 ± 0.009 and control, 0.180 ± 0.014; F1,61 = 6.1, p = 0.016

Locomotor activity and feed consumption

**Fig. 2.**
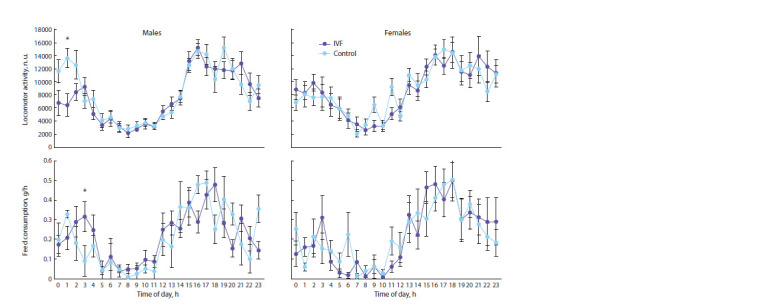
Daily dynamics of locomotor activity and feed intake in progeny obtained by natural conception (Control) or by IVF. * p ≤ 0.05, ANOVA with repeated measure: F1.16 = 5.06 for motor activity and F1.16 = 5.16 for feed intake.

Monitoring of locomotor activity and feed consumption showed typical circadian changes in the studied indices in mice (Fig. 2). Statistically significant differences between the control and experimental groups were found only in males during the second half of the active period, i. e., from 00:00 to 04:00 hours (local time). The control individuals showed an increase in activity, which was statistically significantly higher than that of the IVF progeny, at 01:00 h. In turn, IVF progeny showed higher feed intake than control individuals at 03:00 h.

Analysis of variance with experiment/control and gender factors of the results of 3-day monitoring of locomotor activity and feed consumption showed that spontaneous locomotor activity was independent of whether the animals belonged to the control or experimental (IVF) group (Table 3). For feed intake per 1 g body weight, a significant effect of IVF was found: control individuals consumed more feed (0.630 ± 0.037 g/g, n = 26) than IVF-derived individuals (0.511 ± 0.036 g/g, n = 24). Feed consumption per unit traveled was the same in the control and experimental groups. It should be noted that a statistically significant effect of sex was detected for the studied indicators. At the same time, females showed more spontaneous activity and higher feed consumption compared to males. But feed consumption per unit of the traversed way was 24 % less for them than for males. Statistical significance of sex differences is confirmed by analysis of variance: F1,46 = 10.5, p = 0.0022.

**Table 3.**
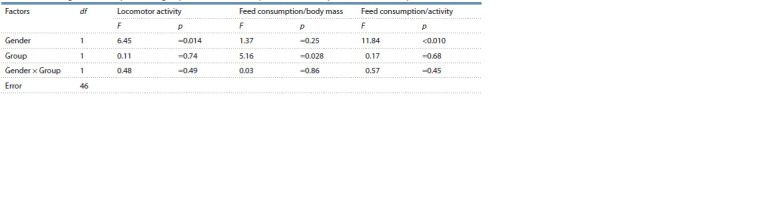
Effects of gender and experimental group on locomotor activity and feed consumption (two-factor analysis of variance)

Glucose tolerance test (GTT)

**Fig. 3.**
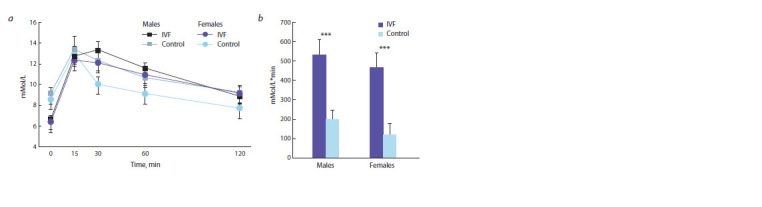
Glucose tolerance test: a, glucose concentration; b, AUC *** p <0.001 between experimental and control offspring groups (Student’s t-test).

The baseline glucose concentration (time 0 in Fig. 3) in males and females obtained by IVF was significantly lower than in controls (Table 4). The maximum values of glucose recorded 15 min after the injections were similar in individuals of different sexes and different experimental groups. The total deviations of glucose concentration (AUC) differed significantly depending on the affiliation with the experimental group (see Table 4). They were statistically significantly higher in IVF progeny than in controls (see Fig. 3).

**Table 4.**
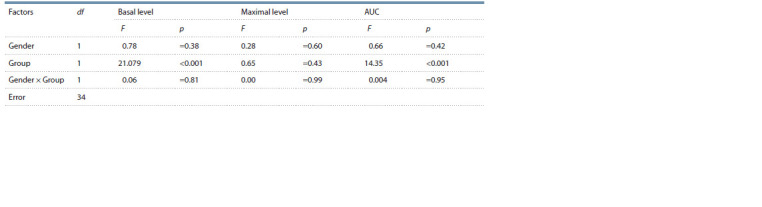
Effects of gender and experimental group on basal and maximal glucose levels and on AUC in the glucose tolerance test (two-factor analysis of variance)

## Discussion

Despite the equalization of the size of the litter in the control and experimental groups, males obtained by in vitro fertilization surpass the body mass of naturally conceived individuals in all age groups. It should be noted that the positive effect of IVF on the growth rate of males is also noted by other authors (Van Montfoort et al., 2012; Donjacour et al., 2014; Narapareddy et al., 2021; Elhakeem et al., 2022). But this effect significantly depends on the conditions of embryo development outside the maternal body. When incubated in a medium with optimized amino acid composition, the body mass of sexually mature IVF males did not differ from controls (Donjacour et al., 2014; Duranthon, Chavatte-Palmer, 2018; Qin et al., 2021) and even exceeded that of females (Feuer et al., 2014).

In contrast to body mass, the effect of IVF on composition was statistically significant in both males and females. The total and relative (% of body mass) fat content was higher in IVF offspring of both sexes. In its turn, IVF males not only had higher body mass compared to control males, but the absolute values of lean mass were higher in them than in control individuals. At the same time, the relative lean mass in control males was superior to that in IVF progeny, at least in females. One metabolically relevant characteristic of body composition is the ratio of total fat mass to lean mass (Seo et al., 2020; Liu et al., 2021). The offspring of both sexes obtained by IVF were 31.6 % (males) and 22.8 % (females) higher in FLR than control individuals.

The most widespread cause of individual variations in fat accumulation is a change in the balance between energy substrate intake and expenditure, particularly for muscular work. Our study revealed no statistically significant differences in the level of spontaneous activity between control and IVF progeny. And feed consumption per unit body mass was lower in IVF offspring than in naturally-raised individuals. Experimental and clinical studies indicate that fat accumulation increases when the main food intake shifts to the end of the active phase of the diurnal cycle (Gill, Panda, 2015; Panda, 2016; Wilkinson et al., 2020). A statistically significant excess of feed consumption 3 h before the end of the dark time of the day was noted in IVF males, which exceeded the control individuals in this indicator. In females of control and experimental groups, the dynamics of feed consumption was the same. But greater fat accumulation compared to the control was characteristic of IVF offspring of both sexes. Therefore, a change in the daily rhythm of feed intake occurring only in males cannot serve as a universal explanation for changes in body composition in IVF offspring

Along with the balance of locomotor activity and feed intake, no less important for fat accumulation is the rate of utilization of energy substrates. The indicator reflecting the rate of utilization of energy substrates can be the drop in blood glucose concentration during a standard carbohydrate load. The area under curve (AUC), the value of which was 2.5 (males) and 3.2 (females) times higher in IVF progeny compared to the corresponding control, served as an integral criterion of the efficiency of the initial glucose level recovery

Therefore, IVF-derived mice differ from the control mice in greater fat accumulation combined with lower feed consumption and reduced tolerance to glucose load. This body composition is in good agreement with the literature, according to which individuals with lower basal metabolic rates are more prone to diabetes mellitus (Maciak et al., 2020). The role of body composition may act as one of the significant factors of the observed metabolic changes. Here, the increase in the ratio of fat to lean mass detected in all sex and age groups of animals draws attention. It is known that adipose tissue makes a minimal contribution to total energy intake, which is largely determined by muscle (Seo et al., 2020; Liu et al., 2021), the main component of lean mass.

To summarize, the results demonstrate that a significant increase in the risk of metabolic syndrome in IVF offspring is independent of the amount of feed consumption and locomotor activity. In males, fat accumulation can be accounted for by impaired daily rhythm of feed consumption and decreased glucose utilization rate. In females, the main cause of fat accumulation and, as a consequence, the risk of metabolic syndrome may be associated with changes in the metabolic pathways that ensure the efficient utilization of energy substrates, and this cause is indicated by a significant decrease in glucose tolerance

It should be noted that our findings indicate possible pathways for the development of the metabolic syndrome, but do not reveal its mechanisms, which may be due to many factors related to the specific effects at different stages of offspring ontogenesis when using the ART complex. In particular, the composition of the culture medium (Khosla et al., 2001; Sjöblom et al., 2005; Zandstra et al., 2018), the oxygen content of the gas medium and the pH of the culture medium (Kelley, Gardner, 2017; Ng et al., 2018), the duration of embryo culture (Johnson, 2019), and other factors associated with surgical embryo transfection procedures (Rozhkova et al., 2017), as well as maternal and fetal immunogenetic differences (Gerlinskaya et al., 2019) affect the phenotype of offspring. Nevertheless, it should be emphasized that these works are limited to the study of individual stages of individual development, which makes it difficult to analyze the cause-effect relationships between successive ontogenetic events. The period of ontogenesis including the first cell division and development of preimplantation embryos is critical and coincides with the global reprogramming of the epigenome and establishment of epigenetic modifications that persist into adulthood. It is likely that epigenetic modifications resulting from exposure to the procedures used in obtaining offspring by ART may play a central role in destabilizing prenatal development and, consequently, in increasing the risk of metabolic syndrome.

One of the criteria used to assess developmental destabilization is fluctuating asymmetry (FA) (Dongen, 2006). The feasibility of using this criterion as an indicator of developmental destabilization is supported by clinical observations showing that FA of fingerprints on the left and right hand with a high degree of reliability is associated with predisposition to diabetes (Morris et al., 2012, 2016; Yohannes et al., 2015).

## Conclusion

Thus, the combination of our own and literature data allows us to outline a range of IVF-conditioned interrelated events that include developmental destabilization, a set of metabolic changes, and increased risk of diabetes. However, the mechanistic specification of the effects of IVF requires further research, including expanded studies of the relationships of epigenetic modifications, fluctuating asymmetry, and metabolic regulation. The relevance of such studies, judging by the data presented in the Norrman review (Norrman et al., 2020), is steadily increasing as the cohort of people born with assisted reproductive technology matures

## Conflict of interest

The authors declare no conflict of interest.
